# Evaluation of the innate immune response of caprine neutrophils against *Mycobacterium avium* subspecies *paratuberculosis* in vitro

**DOI:** 10.1186/s13567-023-01193-7

**Published:** 2023-07-18

**Authors:** Miguel Criado, Valentín Pérez, Noive Arteche-Villasol, Natalia Elguezabal, Elena Molina, Julio Benavides, Daniel Gutiérrez-Expósito

**Affiliations:** 1 Departamento de Sanidad Animal, Instituto de Ganadería de Montaña (IGM) CSIC-ULE, Grulleros, León Spain; 2 Departamento de Sanidad Animal, Facultad de Veterinaria, Universidad de León, Campus de Vegazana s/n, 24071 León, Spain; 3 Departamento de Sanidad Animal, NEIKER-BRTA, Instituto Vasco de Investigación y Desarrollo Agrario, 48160 Derio, Vizcaya Spain

**Keywords:** Neutrophils, NETs, phagocytosis, *Mycobacterium*, paratuberculosis, cytokines, ruminants

## Abstract

Neutrophils constitute an essential component of the innate immune response, readily killing most bacteria through phagocytosis, degranulation, and the release of neutrophil extracellular traps (NETs) among other mechanisms. These cells play an unclear role in mycobacterial infections such as *Mycobacterium avium* subspecies *paratuberculosis* (*Map*), the etiological agent of paratuberculosis, and its response is particularly understudied in ruminants. Herein, a wide set of techniques were adapted, or newly developed, to study the in vitro response of caprine neutrophils after *Map* infection. Immunofluorescence was used to demonstrate, simultaneously, chemotaxis, phagocytosis, degranulation, and NETs. The quantification of neutrophil phagocytic activity against *Map* at a 1:10 multiplicity of infection (MOI), through flow cytometry, showed values that varied from 4.54 to 5.63% of phagocyting neutrophils. By immunofluorescence, a 73.3 ± 14.5% of the fields showed NETs, and the mean release of DNA, attributable to NETosis, calculated through a fluorometric method, was 16.2 ± 3.5%. In addition, the RNA expression of TGF-β, TNF and IL-1β cytokines, measured through reverse transcription qPCR, was significantly higher in the two latter. Overall, neutrophil response was proportional to the number of bacteria. This work confirms that the simultaneous study of several neutrophil mechanisms, and the combination of different methodologies, are essential to reach a comprehensive understanding of neutrophil response against pathogens, demonstrates that, in vitro, caprine neutrophils display a strong innate response against *Map*, using their entire repertoire of effector functions, and sets the basis for further in vitro and in vivo studies on the role of neutrophils in paratuberculosis.

**Supplementary Information:**

The online version contains supplementary material available at 10.1186/s13567-023-01193-7.

## Introduction

Neutrophils are the most abundant leucocytes in blood and are essential for the innate immune response due to their ability to rapidly kill invading pathogens through phagocytosis, the generation of reactive oxygen species (ROS), and the use of antibacterial products stored in their granules [1]. These granules can be fused with the phagosome or with the plasma membrane, in an exocytic process called degranulation, releasing its granule contents to the extracellular space [2]. Additionally, neutrophil extracellular traps (NETs) (a meshwork of chromatin fibers, coated with cytoplasmic granules containing antimicrobial proteins) are able to entrap, immobilize and, in some cases, kill pathogens [3]. Thus, over the past 10 years, the view on the role of neutrophils in the immune response has changed from simple, nonspecific, suicide killers, to complex cells with many specialized functions, capable of shaping the adaptive immune response or even playing a key role in the trained immunity [1, 4].

The relationship between different pathogens and neutrophils has been assessed through the study of different effector mechanisms [1]. Phagocytosis has been traditionally evaluated using manual or automated fluorescence microscopy approaches, but high throughput techniques based on flow cytometry are gaining ground [5]. Degranulation mechanisms have been studied mainly through (i) transmission electron microscopy (TEM), (ii) immunofluorescence [6], (iii) enzyme-linked immunosorbent assays (ELISA) or (iv) fluorometric techniques that evaluate the enzymatic activity of the myeloperoxidase (MPO), lactoferrin, gelatinase or other granule enzymes released in vitro and even through (v) flow cytometric analysis of degranulation surface markers [7]. NETosis has been studied mainly through image-based techniques [3, 8]; however, an extensive repertoire of methods such as ELISA and fluorometric tests has been developed for the evaluation and quantification of NET components [9]. In addition, another interesting aspect of neutrophil function is the expression and production of cytokines that can be measured through qPCR and ELISA, respectively. Although the amount of cytokines produced by neutrophils is lower than by mononuclear cells, their role can be significant [10].

The development of all these techniques has been carried out mainly in human and murine neutrophils [11] and, to a lesser extent, in ruminants such as cattle, but to study its response to nutritional or physiological changes and to extracellular pathogens like protozoan parasites [12, 13].

Concerning mycobacterial infections, the role of neutrophils in the immune response is still poorly understood. Experimental findings regarding the ability of neutrophils to kill pathogenic *Mycobacterium* species such as *M. tuberculosis* and *M. leprae* in vitro are often contradictory. While some studies suggest that neutrophils have impaired capability to kill these pathogens, inhibiting their function in vivo has been shown to exacerbate disease pathology [14]. Other studies show that *M. bovis* readily multiplicate inside bovine neutrophils in vitro [15], whereas in similar in vitro conditions, neutrophils kill *M. avium* subspecies *paratuberculosis* (*Map*) [16]. *M. smegmatis* and *M. leprae* induce neutrophil granule exocytosis, in both cases with negative clinical consequences [17, 18]. *M. tuberculosis*-induced NETs have been previously described in vitro [19] and in vivo [20], while neutrophil NETosis against *Map* has been described very recently in vitro, in rabbit and bovine experimental models [16, 21], although no significant NETosis was observed when ovine neutrophils were infected with *Map* [22]. An exonuclease (MAP3916c), capable of degrading PMA-induced NETs, and promote *Map* spread, has been very recently identified in *Map*-K10 [23]. Regarding cytokine production, it has been shown that less pathogenic *Mycobacterium* species induce the production of IL-6, IL-8 and TNF, suggesting that they may initiate the innate immune response, capable of eliminating this bacterium [24]. Thus, neutrophil function in mycobacterial infections remains unclear. Recent research on neutrophil heterogeneity and its influence on adaptive immunity further complicate this situation. Most research has been done on human tuberculosis where the actual consensus is that neutrophils seem to kill or influence other cells to kill *M. tuberculosis* at the initial stage of the disease while causing a detrimental effect at advanced stage [25].

Paratuberculosis, caused by *Map*, is a widespread mycobacterial infection of ruminants, responsible for important economic losses; however, its impact in goats has not been precisely estimated, even though it is globally distributed in this species [26]. Despite its relevance, its pathogenesis and, particularly, the host immune response, are not fully understood. Host immune response against *Map* is highly variable between individuals and only a portion of the infected animals develop clinical disease in both natural and experimental infections. Most of the information on the local immune response has been gathered from clinically infected animals, and frequently at the endpoint of the experiment. This prevents us to know what happens locally at very early stage of the infection [27], where neutrophils have been proposed to play an important role. In this sense, the presence of neutrophils in the ileum at the initial phase of the disease has been found [28], with an increase in the number of neutrophils present in the lamina propria, crypts, and lumen of the ileum during the first hours of *Map* infection, but their role was not clarified. Other studies in sheep have also found neutrophils as forming part of the early granulomas in lambs infected with *Map* [29]. However, it is thought that they tend to disappear at later stage of the disease, since ileal tissues from clinically infected animals contain less neutrophils than non-infected animals [30]. Additionally, some recent transcriptomic studies performed on animals at late stage of the infection, suggest an impairment of neutrophil recruitment and activation [31, 32]. None of these studies respond to the hypothesis of whether neutrophils could play a role in the clearance of the bacteria at the initial stage of *Map* infection or, if they fail, could facilitate the infection progression. Thus, the role of neutrophils in mycobacterial infections, particularly against *Map*, needs to be further explored. As a natural host of *Map*, goats constitute an excellent experimental model, offering significant advantages for the study of peripheral blood immune cells given its easy management, relatively low maintenance cost and the easy access to large volumes of blood. Additionally, the in vitro approach allows us to gather abundant data on the neutrophil response against *Map*, and it could be applied to study the dynamics of this response within an individual, throughout extended periods of time, and the effects that age, vaccination, or infection could have on it.

Therefore, the aim of this study was to assess the response of neutrophils after in vitro infection with *Map*, with the final objective of achieving a better understanding of the innate response to these bacteria and laying the foundation for further studies with other *Mycobacterium* species. For this purpose, an isolation protocol was developed to preserve caprine neutrophil functionality and viability. Adequate techniques based on immunofluorescence, fluorometry, scanning electron microscopy (SEM), flow cytometry and qPCR were also developed or adapted from other animal species for the in vitro study of the innate response against *Map.*

## Materials and methods

### Ethics statement

Goat handling and blood sample collection were carried out in accordance with European Union legislation (Law 6/2013) concerning animals, their exploitation, transportation, experimentation and sacrifice; R. D. 118/2021 for the protection of animals employed in research and teaching; Directive 2010/63/UE, related to the protection of animals used for scientific goals. All the procedures were approved by the corresponding animal welfare body (OEBA) and the Consejería de Agricultura y Ganadería de la Junta de Castilla y León (authorization code ULE-02-2021). All animals used in this study were handled in strict accordance with good clinical practices, and all efforts were made to minimize suffering.

### Animals and experimental design

Three healthy 8-month-old male goats randomly selected from a commercial flock without previous history of paratuberculosis were used in this study. All animals tested negative for paratuberculosis antibody ELISA using the commercial kit ID Screen® Paratuberculosis Indirect (IDvet, Gabrels, France) and for the interferon-γ release assay (BOVIGAM™ TB Kit, Thermo Fisher Scientific, Waltham, USA).

Blood samples were collected to isolate neutrophils from each animal and to evaluate the in vitro response against *Map* through different approaches. Specifically, immunofluorescence and SEM were used for the visualization of chemotaxis, phagocytosis, degranulation and NETosis. Phagocytosis was quantified through flow cytometry, while quantification of NETs was carried out through both immunofluorescence and indirect fluorometric methods. In addition, transcript expression of cytokines was evaluated through qRT-PCR.

Briefly, after isolation, neutrophils were exposed to both live and heat-killed *Map* (k*Map*) at two different multiplicities of infection (MOI) of 1:1 and 1:10 [33] and at different time points. Live *Map* and k*Map* containing an expression plasmid with green fluorescent protein-GFP [16, 21] were used for immunofluorescence and flow cytometry. In addition, two different non-bacterial inducers were used: (i) phorbol 12-myristate 13-acetate (PMA) (Sigma-Aldrich, St. Louis, MO, USA) at a final concentration of 50 nM, and (ii) Zymosan A (Sigma-Aldrich) at a final concentration of 1 mg/mL, as previously described [16]. Negative controls (non-infected and non-stimulated neutrophils) were also included for every animal in all assays.

### Neutrophil isolation

Neutrophils were isolated from heparinized blood using a previously described, density gradient method, based on Lymphoprep® (STEMCELL Technologies®, Grenoble, France) [34].

Twenty five mL of whole blood were diluted in 25 mL of PBS and centrifuged at 650 *g* for 30 min at 21 °C, the buffy coat was collected, resuspended in 20 mL of PBS, layered on 20 mL of Lymphoprep®, and centrifuged at 650 x *g* for 30 min at 21 °C, the mononuclear cell layer was discarded and the granulocyte layer on top of the erythrocyte pellet was transferred to another tube, erythrocyte lysis was performed with 0.03% sodium bicarbonate and stopped with PBS at 4 °C (this step was performed twice if remaining erythrocytes were observed). After centrifugation at 300 x *g* for 10 min at 4 °C, the pellet was washed with PBS, centrifuged at 300 x *g* for 10 min at 4 °C and resuspended in incubation medium (explained below).

Four modifications were made on the cited [34] isolation protocol, first, PBS was used without EDTA as it is known to inhibit NET formation [35], and calcium is required for degranulation [36]. Second, erythrocyte lysis was performed using 3 mL of 0.03% sodium bicarbonate for 10 s, and PBS was added up to 50 mL. Third, neutrophils were kept at 4 °C from the erythrocyte lysis step. Fourth, Gibco™ RPMI 1640 (11835-063, Thermo Fisher Scientific, Waltham, MA, USA) medium was used without phenol red and supplemented with 2% heat-inactivated fetal bovine serum (10500064, Gibco®, Paisley, UK) and L-glutamine (25030081, Gibco®, Paisley, UK) at a final concentration of 2mM.

Neutrophils were counted in a Neubauer chamber and used for the assays included in this study, a high viability (> 98%) and purity (> 90% of neutrophils, with the remaining cells being eosinophils) of the final product was achieved as determined respectively by Trypan blue (Gibco®, Paisley, UK) exclusion test and Diff-Quik™ staining, flow cytometry scatter parameters [34] and MPO labelling, using the technique explained below.

### Bacteria preparation

*Map* K10 strains (with and without a GFP expression plasmid) were grown to exponential phase for 3 weeks at 37 ± 1 °C, in 7H9 broth supplemented with 10% oleic acid-albumin-dextrose-catalase enrichment (OADC) (Becton Dickinson and Company, MD, USA), 0.2% glycerol, 0.05% Tween 80 (Panreac Quimica SA, Barcelona, Spain) and 2 mg/L of mycobactin J (IDVET, Gabrels, France) (7H9-OADC-MJ). *Map* K10-GFP was grown on the same media supplemented with kanamycin (25 µg/mL) (7H9-OADC-MJ-Kan). Bacterial growth was estimated by optical density at 600 nm and based on data curves and plating assuming that 0.7 OD600 is 1 × 10^8^ bacteria/mL for *Map*.

Bacterial suspension was adjusted to a concentration of 10^8^*Map* CFU/mL in glycerol: water (1:1) after colony forming units (CFUs) estimation by optical density and colony count in agar-solidified 7H9 with OADC, glycerol and mycobactin J to assess the CFUs per mL in the inoculum. Afterwards, bacterial suspension was frozen at − 80 °C until use.

*Map* aliquots were thawed and resuspended in fresh 7H9-OADC-MJ or 7H9-OADC-MJ-Kan and incubated for 3 h at 37 °C. Then, bacterial suspensions were centrifuged at 5000 x *g* for 10 min and *Map* pellets were washed twice with PBS and a half of this aliquot was heat-inactivated (85 °C, 30 min) [16]. After that, both live and inactivated bacteria were resuspended in cell culture media, passed through a 27-gauge syringe needle and vigorously vortexed to disperse clumps before in vitro infection.

### Immunofluorescence

A total of 5 × 10^5^ neutrophils were seeded in duplicate on 13 mm ø sterile poly-l-lysine (0.01%) (Sigma-Aldrich) pre-coated cover glasses in 24-well plates [13]. Incubation was performed at 37 °C in a 5% CO_2_ atmosphere, and a 3 h incubation time was selected based on previous studies that used a range between 1 and 4 h of incubation [3, 8]. Neutrophils were incubated with the different inductors and MOIs of *Map* mentioned above (see sub-section “Animals and experimental design”). Afterwards, cells were fixed with methanol-free paraformaldehyde (Thermo Fisher Scientific, Waltham, MA, USA) up to a final concentration of 4%. Then, permeabilization and blocking were performed with 0.25% Triton™ X-100 (Sigma-Aldrich) and 3% bovine serum albumin (BSA) (Roche Diagnostics, Mannheim, Germany) in PBS for 1 h at 37 °C. After two washes with PBS, an overnight incubation with both the rabbit anti-MPO polyclonal Alexa Fluor 750 Conjugated (AF750) (BS-4943R-A750, Bioss, Woburn, MA, USA) [37] at a 1:200 dilution, and the mouse anti-pan-histone primary antibody (MAB3422, Merck, Darmstadt, Germany) [16] for NETs detection, at a 1:400 dilution was performed. Then, for the visualization of histones, samples were incubated for 1 h with the secondary antibody goat anti-mouse IgG (H + L) cross-adsorbed AF647 (Invitrogen, Carlsbad, CA, USA). Animal-free blocker® and diluent R.T.U. (Vector) was used for antibody dilutions. Finally, cover slips were mounted on slides using Fluoroshield™ with DAPI (4',6-diamidino-2-phenylindol) (Sigma-Aldrich). Images were taken using the direct microscope Eclipse Ni-E (Nikon) equipped with the Prime BSI Scientific CMOS scientific camera (Photometrics® Prime BSI™, Scottsdale, AZ, USA).

### Scanning electron microscopy (SEM)

For SEM analysis, neutrophils were incubated without stimuli and with live *Map* at a 1:10 MOI in the same conditions as for immunofluorescence microscopy (see sub-section “Immunofluorescence”). After incubation, cells were fixed in 2.5% EM grade glutaraldehyde (R1012, Agar Scientific, Stansted, UK), post-fixed in 2% osmium tetroxide aqueous solution (Electron Microscopy Sciences, Hatfield, PA, USA), washed in PBS, dehydrated using a graded ethanol series, critical point dried by CO_2_-treatment (Leica EM CPD300, Leica Microsystems, Wetzlar, Germany) and sputtered with gold particles (Leica EM ACE200, Leica Microsystems, Wetzlar, Germany). Finally, all samples were visualized through the JSM-6480LV (JEOL, Tokyo, Japan) scanning electron microscopy with the software SEM control user interface version 7.60 (JEOL, Tokyo, Japan).

### NETosis quantification

For the quantification of NETosis, two different approaches were used, one based on the visualization of NETosis through fluorescence microscopy using an antibody against histones, and another based on the fluorometric quantification of DNA release [12, 21].

First, all the cover slips used for visualization of NETs (see sub-section “Immunofluorescence”) were also used to perform an estimation of NETosis. Briefly, a total of 10 random fields (400×), with equal neutrophil densities (97 neutrophils/field on average) were analyzed per slide of each animal, and a field was considered as positive if at least one neutrophil was clearly undergoing NETosis, identified by the release of both DNA and histones in a branching pattern. Microphotographs were taken using the Eclipse Ni-E (Nikon, Tokyo, Japan) microscope, with the DS-Ri2 color microscope camera (Nikon, Tokyo, Japan).

Second, for quantifying the DNA release, 2 × 10^5^ neutrophils were seeded in duplicate in 96-well plates and incubated in the same conditions, using uninfected neutrophils as controls and the same stimuli and *Map* MOIs for stimulation employed in the immunofluorescence assay (see sub-section “Immunofluorescence”), in a final volume of 100 µL. After 3 h incubation, 10 gel units of *Staphylococcus aureus* micrococcal nuclease (New England Biolabs, Ipswich, MA, USA) were added for 15 min at 37 °C. The reaction was stopped by adding EDTA up to a final concentration of 5 mM in a total volume of 200 µL. The plate was then centrifuged for 15 min at 300 x *g* and a half of the final volume (100 µL) was transferred to another plate. In addition, extracted DNA using the Maxwell® RSC Cultured Cells DNA Kit (Promega) from an equal number of non-stimulated neutrophils of each animal was eluted in 200 µL of nuclease-free water and divided into two wells. Later, 100 µL of Quant-iT PicoGreen dsDNA Reagent (Invitrogen, Carlsbad, CA, USA), diluted 1:200 in TE buffer was added to each well, and incubated for 5 min protected from light. Finally, fluorescence intensity was measured using the automated microplate reader Biotek® Synergy HT (Biotek Tecnologies, Santa Clara, CA, USA). Extracellular DNA concentration was calculated using the standard curve of Lambda DNA provided by the kit.

To compensate background fluorescence, autofluorescence and DNA released by spontaneous NETosis and cell death, the mean DNA concentration of the non-stimulated neutrophils from each animal, was subtracted from the mean DNA concentration of each sample. Afterwards, the percentage of released DNA of each sample, was calculated in relation to the mean total DNA extracted from the same number of neutrophils from the same animal [21].

### Neutrophil *Map* phagocytosis assay

A total of 2.5 × 10^6^ neutrophils from each animal were infected with *Map*-GFP at a MOI of 1:10 in a final volume of 1 mL. A negative control without *Map* was included for each animal. Both infected and controls were incubated in a tube rotator (Fisherbrand^™^, Waltham, MA, USA), at 37 °C, an angle of 75 degrees and a speed of 6 rpm for 20 min. A time of 20 min incubation in suspension was selected according to previous studies on *Map* and *S. aureus* [5, 21]. An additional negative control was kept at 4 °C without being rotated.

Afterwards, all samples were constantly kept at 4 °C, fixed in 2% paraformaldehyde for 10 min, permeabilized with 70% ethanol for 10 min and resuspended in PBS with 1% BSA. Half of the volume was kept at 4 °C as non-labelled control for further adjusting the autofluorescence threshold of each sample and the other half was incubated for 30 min with the rabbit anti-MPO polyclonal antibody AF750 (BS-4943R-A750, Bioss, Woburn, MA, USA) at a 1:50 dilution, washed twice and resuspended in PBS with 1% BSA.

**Figure 1:**
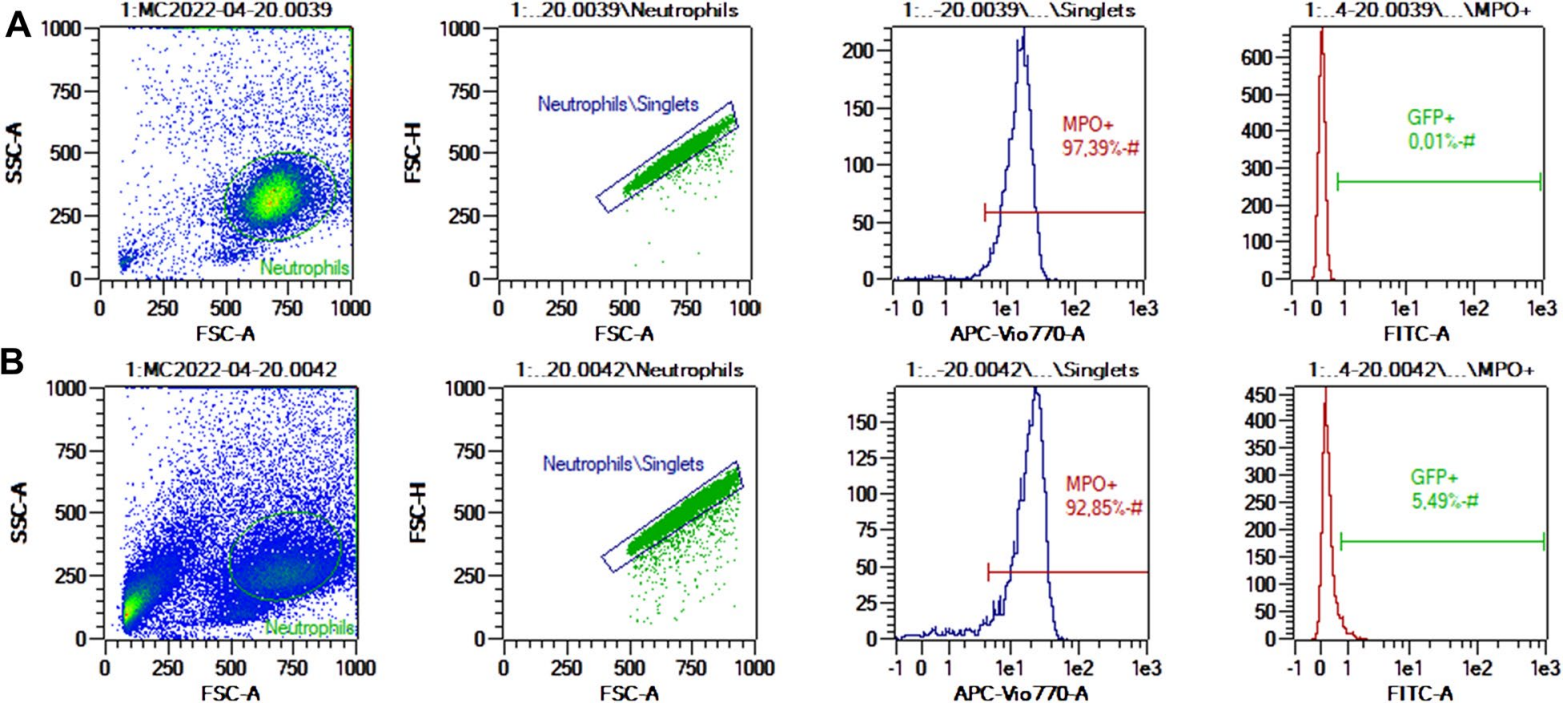
**Gating strategy used for phagocytosis quantification.** **A** Neutrophils, **B** Neutrophils with *Map*-GFP. In the first column, cells were gated based on FSC and SSC; in the second column, a doublet discrimination strategy was applied; in the third column, neutrophils were gated by myeloperoxidase positivity by fluorescence in the APC-Vio770 channel; in the fourth column, GFP + neutrophils were gated in the FITC channel and considered positive to phagocytosis of *Map-*GFP. Uninfected, non-incubated cells were used to ensure cell integrity and purity. Uninfected, incubated cells were used to adjust the neutrophil gate based on scatter parameters. This sample was also used to adjust the fluorescence thresholds of GFP and AF750. Flow cytometry data from 10 000 neutrophils from each animal (*n* = 3) was analyzed using MACSQuantify™ software (Miltenyi Biotec Inc).

Afterwards, flow cytometry data was acquired using MACSQuant® Analyzer 10 flow cytometer (Miltenyi Biotec, San Diego, CA, USA). Gating strategy is detailed in Figure 1.

### Analysis of cytokine expression by qRT-PCR

The mRNA expression levels were determined by quantitative real time PCR (qRT-PCR) as described elsewhere [38]. Primer sequences used for IL-1β, TNF and TGF-β and the three reference genes β-actin, succinate dehydrogenase complex subunit A (SDHA) and glyceraldehyde 3-phosphate dehydrogenase (GADPH) have been previously described (Additional file 1) [38–41].

A total of 2.5 × 10^6^ neutrophils were seeded in 6-well plates and incubated with live and k*Map* at a MOI of 1:1 and 1:10, and with 50 nM, PMA, at 37 °C in a 5% CO_2_ atmosphere. After 6 h of incubation, supernatants were discarded, and neutrophils were washed with PBS and collected for RNA extraction. Briefly, total RNA isolation from neutrophils was carried out using the Maxwell® 16 LEV simplyRNA Cells Kit and with the Maxwell® 16 Instrument (Promega), following manufacturer’s instructions. The RNA was quantified using a QuantiFluor™ RNA System kit and Quantus™ Fluorimeter (Promega, Madison, WI, USA), following manufacturer’s instructions whereas RNA purity was assessed using a NanoDrop 1000 (Thermo Fisher Scientific, Waltham, USA), with the acceptable 260/280 absorbance ratio set to 1.7. Then, reverse transcription to cDNA up to a total of 2500 ng was performed using SuperScript™ VILO™ Master Mix (Invitrogen, Carlsbad, CA, USA), according to the manufacturer’s instructions using SimpliAmp™ Termal Cycler (Applied Biosystems™, Warrington, UK). Finally, cDNA samples were adjusted to 1 ng/µL by dilution in nuclease-free water and stored at − 80 °C until use.

PCR reactions were performed in a 96-well plate (Applied Biosystems™, Warrington, UK) using 10 µL of PowerUp™, SYBR™ Green master mix (Applied Biosystems™, CA, USA), 10 µM of each primer and 2 µL of diluted cDNA template on a 7500 Fast Real-Time PCR System (Applied Biosystems™, CA, USA). Amplification efficiencies were analyzed including a seven-point standard curve for each target gene on every plate prepared from 10-fold serial dilutions of a starting concentration of 1 ng/µL of a conventionally prepared PCR product. All cDNA samples were prepared in parallel and analyzed on the same time.

Data were analyzed using the 2^−ΔΔCt^ relative quantification method as previously described [42]. Briefly, to assess the effect of the different stimuli on neutrophil cytokine expression, for each problem sample, the ΔCt value (gene of interest Ct—mean Ct of the reference genes) was calculated, the ΔCt value of non-stimulated neutrophils (control sample) from the same animal was calculated in the same manner, and used as a calibrator to calculate the ΔΔCt (problem sample ΔCt - control sample ΔCt) of each transcript, then the fold change (FC) in gene expression was calculated (2^−ΔΔCt^) and a log transformation (log_2_FC) was performed to avoid skewness, achieving a symmetrical distribution of the data around 0, in order to better analyze and visualize it [43].

### Statistical analysis

Given the small number of individuals and samples, data were analyzed using non-parametric tests. The Wilcoxon signed-rank test was conducted to determine if the change in MPO mean fluorescence intensity between GFP + neutrophils with respect to the GFP- neutrophils in each sample was significative. For the rest of the data, Kruskal-Wallis and *post hoc* Dunn’s test, with Benjamini-Hochberg correction was used to perform pairwise comparisons between the different stimuli. *P*-values < 0.05 were considered statistically significant. All statistical analyses were performed with the R software version 4.1.3 [44].

## Results

### Visualization of NETs and degranulation

**Figure 2:**
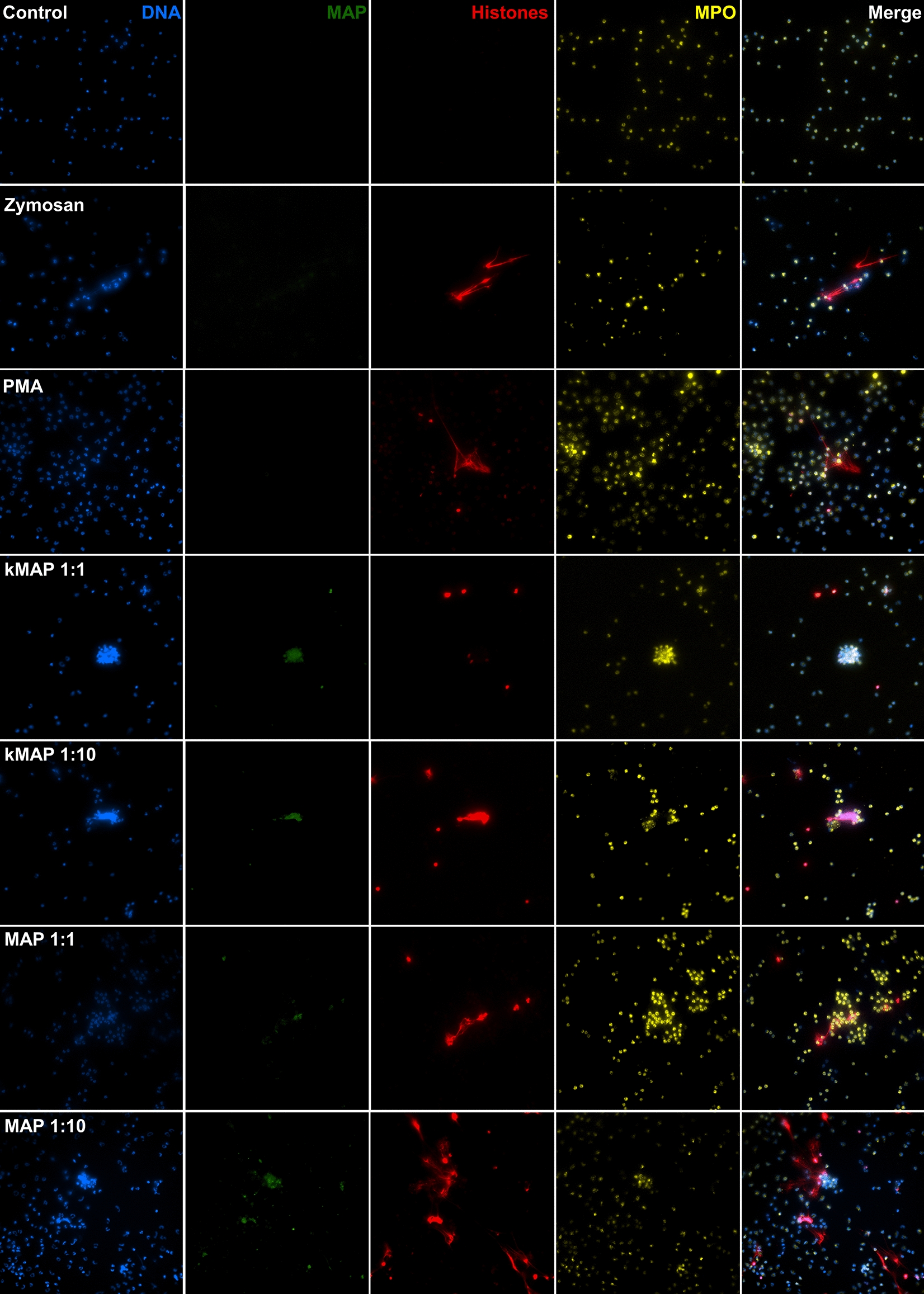
**Immunofluorescence staining of neutrophils after 3 h incubation**. Non-infected, unstimulated neutrophils (Control), with Zymosan, PMA (phorbol myristate acetate), heat-inactivated *Map* (kMAP) and live *Map* (MAP) at 1:1 and 1:10 MOI. DNA (blue), *Map*-GFP (green), histones (red), myeloperoxidase (yellow). Micrographs were taken at 400x using the Eclipse Ni-E (Nikon) microscope and the Prime BSI Scientific CMOS scientific camera (Photometrics® Prime BSI™). Representative fields (*n =* 3, two technical replicates per animal).

Negative controls showed a homogeneous distribution of neutrophils through the slide, exhibiting uniform nuclei shape and size, and MPO + granules seemed to occupy most of the cytoplasm, which led to an inverse staining pattern to that of the DNA (Figure 2 Control). Only a small number of nuclei were labelled for histones and, in some fields, solitary neutrophils were apparently releasing very short and linear extracellular traps.

Neutrophils stimulated with Zymosan A and PMA showed NETs which could be seen in most fields; however, clear differences between both inducers in terms of shape, size, and number of neutrophils involved, were observed (Figure 2 Zymosan and PMA). Specifically, NETs induced by Zymosan A were less abundant than those induced by PMA, and generally unidirectional, with one to three thick branches involving a small number of neutrophils. By contrast, NETs induced by PMA frequently showed a delicate, intricate branching, which embraced a broader area with many neutrophils involved. In both cases, the nuclei of those cells which were not undergoing NETosis, kept their shape and size. However, the intracellular distribution of MPO + granules changed so that only some of the neutrophils stimulated with Zymosan A showed an increase in size, with the granules evenly distributed through the cytoplasm, leading to a duller labelling, while the rest had a compact appearance. Neutrophils stimulated with PMA were uniformly expanded, a feature which could be clearly seen already by light microscopy, and the MPO + granules were evenly distributed through the cytoplasm up to the point that most granules could be individually differentiated. Also, extracellular MPO + granules could be observed in most fields.

Neutrophils stimulated with k*Map* at a 1:1 MOI (Figure 2 kMAP1:1) showed very low numbers of NETs, generally one or two NETs per field and a MPO + staining similar to that seen in non-stimulated neutrophils. However, neutrophils could be seen forming aggregates around the scarce clumps of bacteria. On the other hand, at a 1:10 MOI of k*Map* (Figure 2 kMAP 1:10), NETs were present in most fields, as well as neutrophil aggregates, sometimes releasing MPO + granules on bacterial clumps. In both cases, *Map*-GFP fluorescence intensity was lower than that observed with live bacteria.

**Figure 3:**
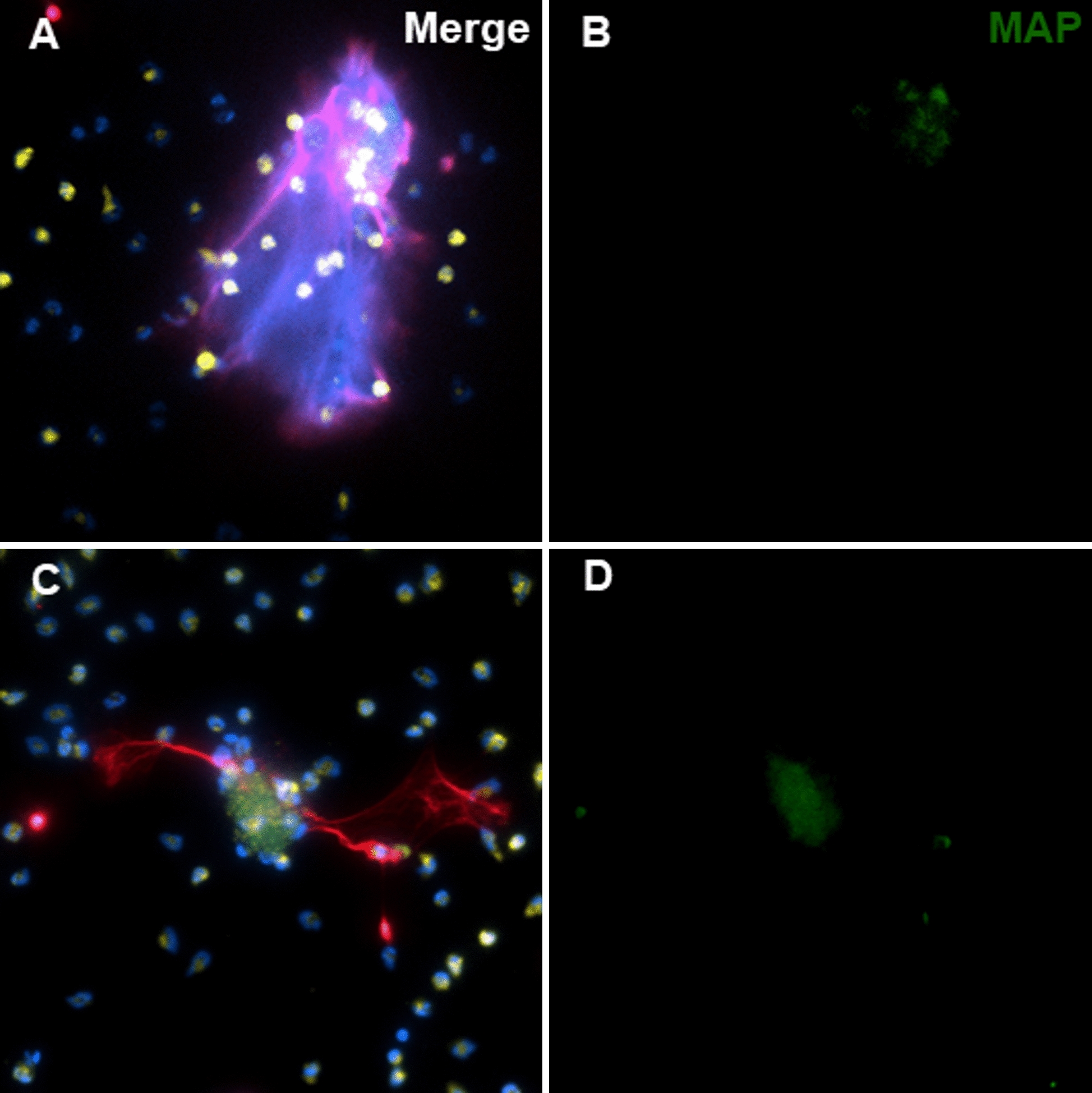
**Neutrophils after 3 h of incubation with live** ***Map*** **at a 1:10 MOI.** Some of the extracellular traps are apparently released by distant neutrophils over bacterial clumps (**A** and **B**), often NETs are released from both, neutrophils in direct contact with bacteria as well as by distant neutrophils (**C** and **D**). DNA (blue), *Map*-GFP (green), histones (red), myeloperoxidase (yellow). Micrographs were taken at 400 × using the Eclipse Ni-E (Nikon) microscope and the Prime BSI Scientific CMOS scientific camera (Photometrics® Prime BSI™).

**Figure 4:**
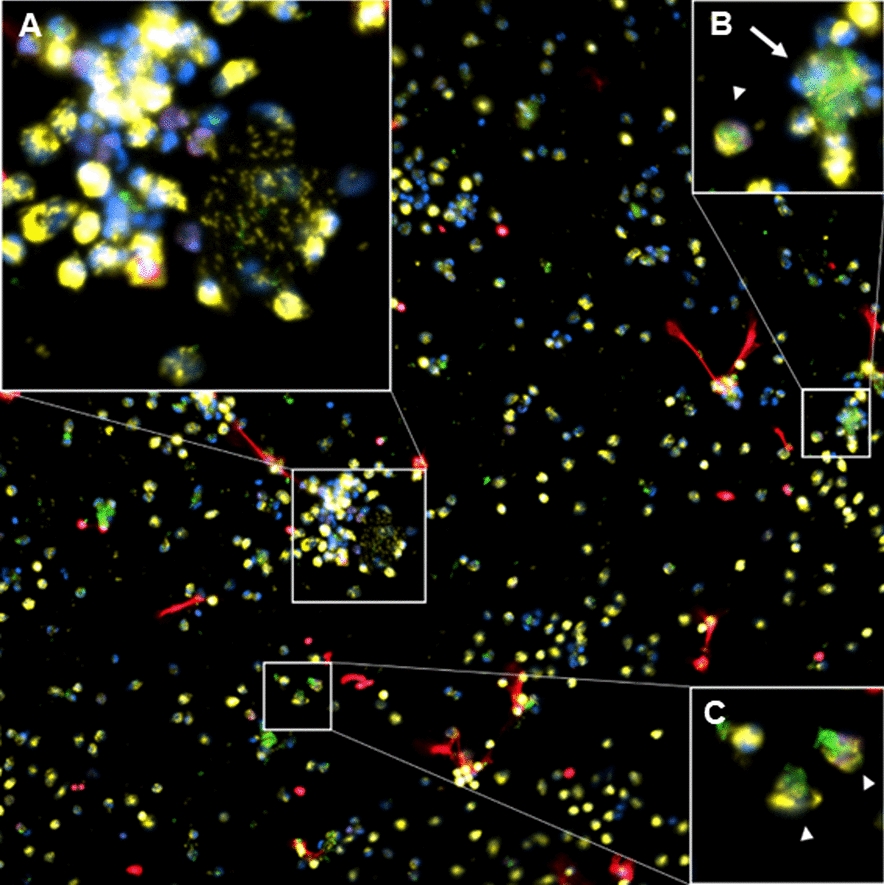
**Neutrophils after 3 h of incubation with live** ***Map*** **at a 1:10 MOI.** Neutrophils often swarm around bacteria (inset **B**, arrow) and release myeloperoxidase (MPO) positive granules on bacterial clumps (inset **A**). Neutrophils with apparently internalized bacteria are frequent (insets **B** and **C**, arrowheads). In this micrograph, several NETs released by small numbers of neutrophils in contact with *Map* can also be observed, as well as a great number of free MPO + granules. DNA (blue), *Map*-GFP (green), histones (red), MPO (yellow). Micrograph was taken at 200 × using the Eclipse Ni-E (Nikon) microscope and the Prime BSI Scientific CMOS scientific camera (Photometrics® Prime BSI™).

Live *Map* at a 1:1 MOI (Figure 2 MAP 1:1) barely affected the neutrophil MPO distribution, though some degranulation could be observed, but the number of NETs was clearly higher than those induced by k*Map* using the same MOI. At a 1:10 MOI (Figure 2 MAP 1:10), NETs could be seen in most fields. In some cases, extracellular traps were apparently formed by the release of DNA and histones over bacterial clumps from distant neutrophils (Figure 3A) while, in other cases, neutrophils in close contact with *Map* clumps, released small NETs as seen in Figure 4, or a combination of both situations (Figure 3C). NETs were always present in the vicinity of the larger *Map* clumps, which were particularly abundant when using live *Map* at this MOI (1:10); additionally, these NETs were large and involved more neutrophils. In addition, neutrophils with variable quantities of internalized *Map* were present in most fields (Figure 4), and neutrophil aggregates surrounding and, sometimes, releasing their MPO + granules over bacterial clumps (Figures 3 and 4) were also common at this MOI.

### SEM microscopy

**Figure 5:**
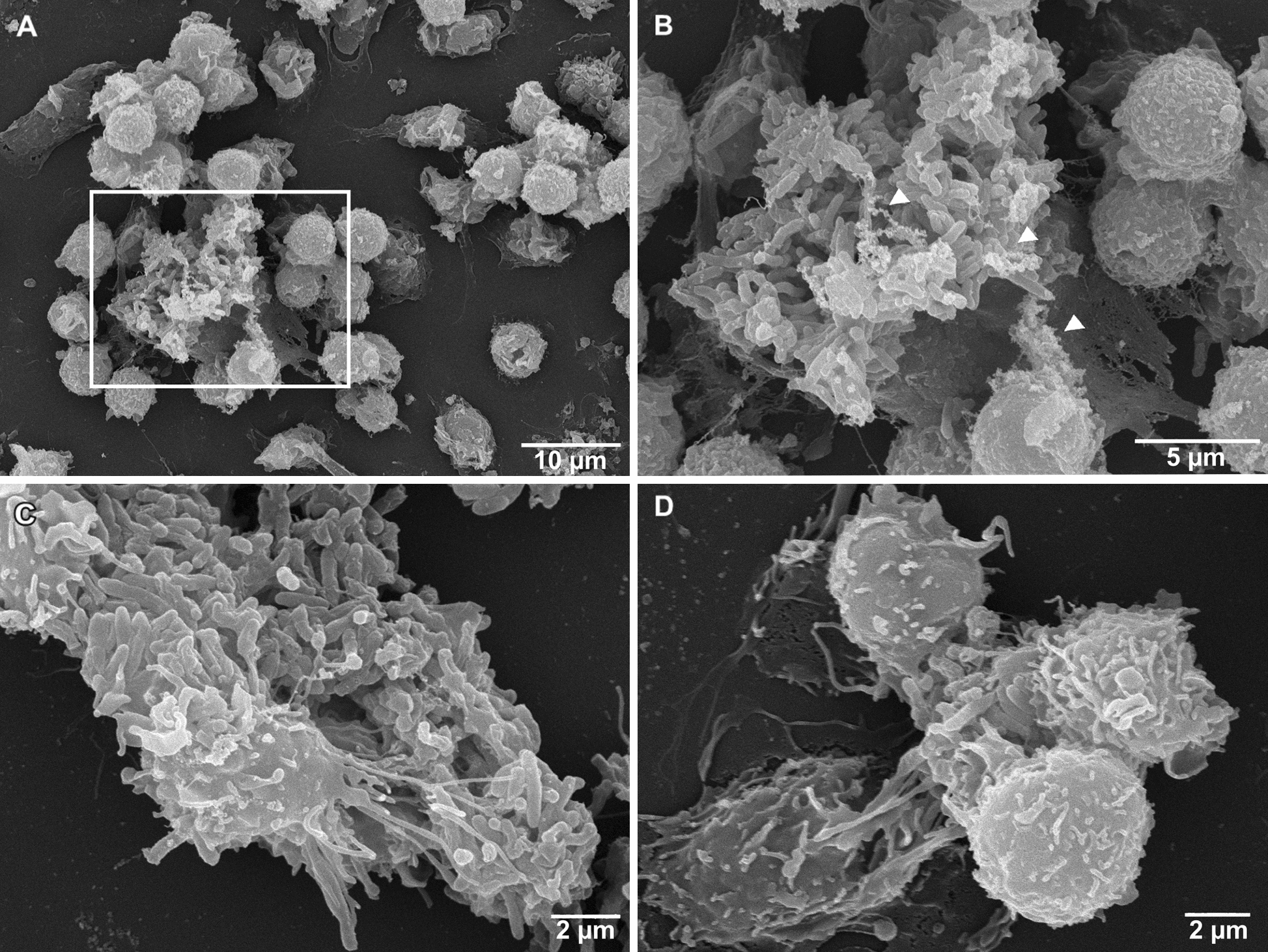
**Results of SEM analysis.** **A** Neutrophil aggregate around a large bacterial clump (center left) and a smaller clump (upper right). **B** Detail of (**A**), delicate NETs (arrowheads) can be seen extruded from some of the neutrophils and amidst the bacilli, where some of them are decorated with small globular domains. **C** Detail of a single neutrophil undergoing NETosis over a bacterial clump. **D** Neutrophils surround a small bacterial clump and release short NETs, the two on the lower right are seemingly phagocytosing bacteria, on the left corner the remnants of an apoptotic neutrophil and NETs can be observed. Representative images (*n =* 3).

SEM analysis revealed that *Map* triggers the formation of neutrophil aggregates (Figure 5A), particularly around the larger bacterial clumps, and the release of strands of variable thickness morphologically consistent with NETs (Figure 5B, C). In addition, neutrophils apparently phagocyting *Map*, as well as apoptotic neutrophils and cellular debris (Figure 5D), could be frequently observed around and over the bacterial clumps. No signs of neutrophil activation could be seen in the non-stimulated neutrophils (Additional file 2).

### NETosis quantification

**Figure 6:**
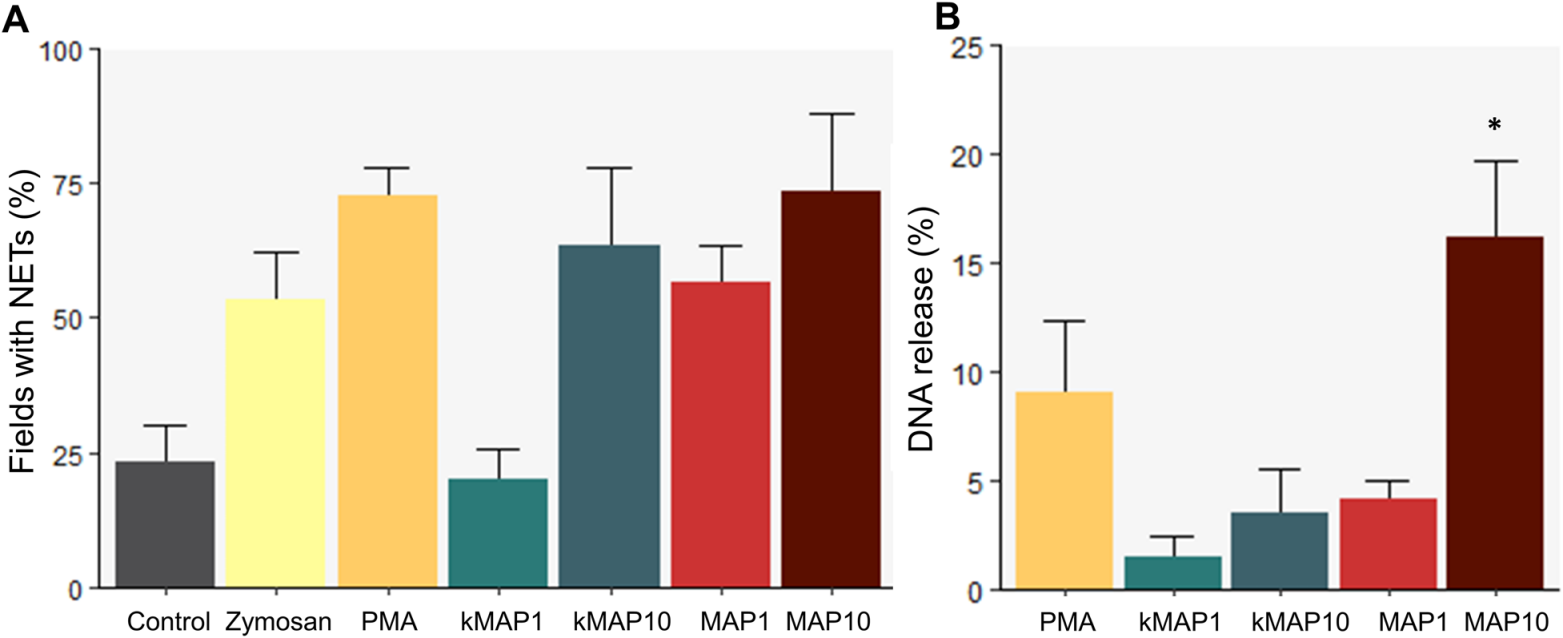
**NETosis quantification**. **A** Percentage of fields (400×) with at least one neutrophil undergoing NETosis for each stimulus. **B** Fluorometric quantification of neutrophil DNA release. All values are means with error bars representing the standard error. Asterisks represent significant differences with the control group. * *p* < 0.05 (*n =* 3, two technical replicates per animal).

NETosis quantification values and statistical differences are shown in Figure 6. The highest number of fields with NETs was seen under PMA and 1:10 live *Map* stimulation, where the percentage of positive fields was higher, approaching significancy (73.3% ± 14.5%, *p* = 0.08; and 65% ± 7.6%, *p* = 0.08, respectively) than that of untreated neutrophils (23.3%% ± 11.5%). However, k*Map* at a 1:10 MOI, live *Map* at a 1:1 MOI and Zymosan A did not cause significant increases in the number of positive fields.

Regarding the DNA release calculated by fluorometry, live *Map* at a 1:10 MOI showed the highest level of DNA release (16.15% ± 3.5%, *p* < 0.05) (Figure 6). PMA stimulation caused a non-significant increase in DNA release (9 ± 3.3%) whereas both MOIs of inactivated *Map*, together with live *Map* at a 1:1 showed the lowest DNA release levels.

The mean DNA release of neutrophils stimulated with Zymosan A was close to 100% (86 ± 33%). Thus, we also quantified the DNA concentration from the supernatants of wells incubated in the same conditions, using the same media and concentration of positive controls, but not seeded with neutrophils, and wells with only Zymosan A, obtaining a higher concentration (2023.63 pg/mL), than the mean total DNA concentration from neutrophils (1875.89 pg/mL). In contrast, supernatants from wells with only PMA yielded a 60.13 pg/mL DNA concentration, attributable to autofluorescence from media components.

### Phagocytosis assay

The flow cytometry analysis revealed that *Map*-GFP caused a significant change in neutrophil size and granularity (Figure 1). In addition, the percentage of neutrophils with internalized *Map*-GFP for each animal was: 5.41%, 5,49% and 4.5% (Mean: 5.13 ± 0.55%), and the mean GFP fluorescence intensity of those positive cells was 1.46, 1.23 and 1.12 (Mean: 1.27 ± 0.17). In addition, GFP + neutrophils showed a non-significant decrease − 39.26%, − 30.06% and − 16.19% (Mean: -28.5% ± 11.61%, *p* = 0.064) in MPO fluorescence intensity.

During the adaptation of this technique, bacterial clumps showed scatter parameters, like those of neutrophils, entering the same gate, and therefore affecting the apparent phagocytosis percentage; however, this inconvenient disappeared after introducing the ethanol permeabilization step required for the intracellular labelling of MPO. Also, regarding permeabilization, we initially tried a permeabilization with 0.25% Triton™ X-100, which was discarded as it caused a highly significant alteration in scatter parameters and cell loss during the preparation steps prior to flow cytometry (data not shown).

### Differential transcript expression of TNF, IL-1β and TGF-β

**Figure 7:**
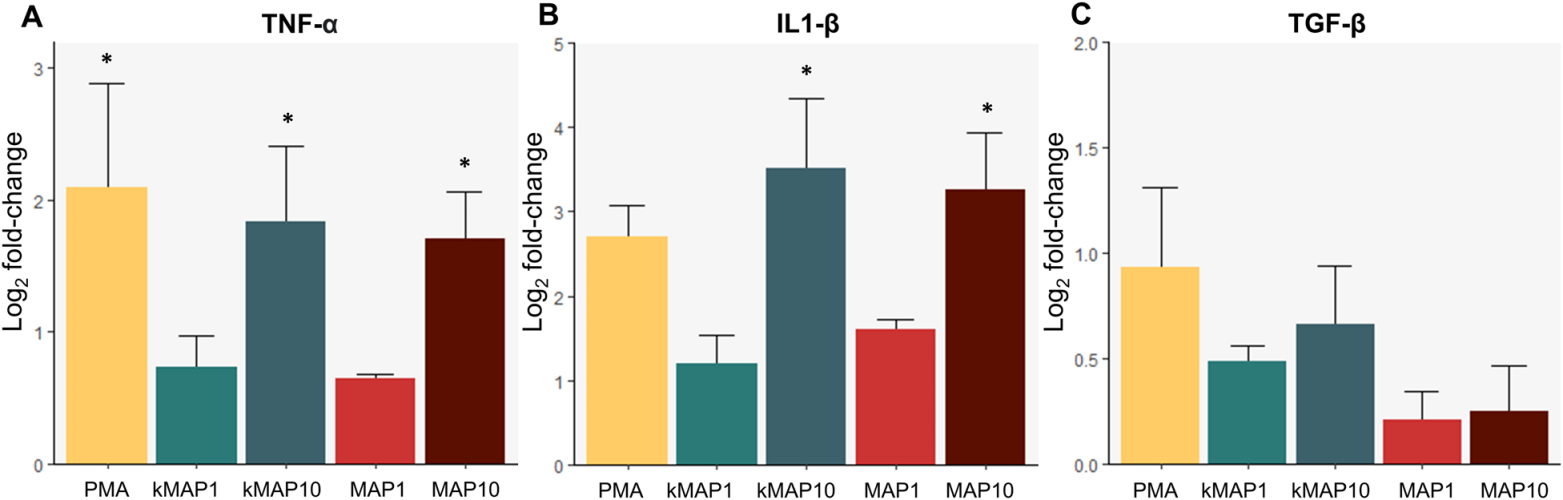
**Cytokine transcript expression.** Bar plots represent the mean of the log_2_-fold change values determined by qRT-PCR in **A** TNF, **B** IL-1β and **C** TGF-β gene expression with error bars representing the standard error. Asterisks represent significant differences with the control group. * *p* < 0.05 (*n =* 3, two technical replicates per animal).

The changes in the relative mRNA expression of TNF, IL-1β and TGF-β of the infected or stimulated neutrophils are represented in Figure 7. None of the RNA samples showed a significant decrease in the β-actin gene expression, suggesting equivalent RNA integrity and the suitability of this gene as internal control. Significant increases in the expression of proinflammatory cytokines with respect to the untreated neutrophils were as follows, for IL-1β: live *Map* at a 1:10 MOI (3.2 ± 0.67; *p* < 0.05) and k*Map* at a 1:10 MOI (3.51± 0.82; *p* < 0.05); and for TNF: PMA (2.09±0.78; *p* < 0.05), live *Map* at a 1:10 MOI (1.71±0.35; *p* < 0.05) and inactivated *Map* at a 1:10 MOI (1.84±0.57; *p* < 0.05) (Figure 7). However, no significant differences were found in the expression of TGF-β in response to any of the stimuli.

## Discussion

The role of neutrophils in mycobacterial infections in ruminants is still unclear despite of the importance of paratuberculosis in livestock. The few studies on ruminant neutrophils and, specifically in goats, have mostly centered its attention in few of its effector mechanisms, offering a partial vision of its role. Here, we have used several different and complementary in vitro techniques to study the main mechanisms employed by caprine neutrophils against pathogens, offering spatial and comprehensive information, and giving a wider and more accurate point of view, increasing the robustness of any finding. Interestingly, in vitro studies on neutrophils require primary cells to study their functions, since immortalized cell lines (e.g. HL-60) present numerous disadvantages like limited antimicrobial activity [45], impaired chemotaxis and swarming [46], lack of some of the specific granules [47] or inefficient generation of NETs [48]. However, primary neutrophils have a very short lifespan, estimated by most authors in less than 24 h, and are extremely sensitive to in vitro studies [49]. Because of that, very refined protocols and the use of appropriate inducers as positive controls are required, as even slight variations in the techniques can thwart neutrophil function or lead to its “spontaneous” activation [8]. This is particularly important given the sensitivity of these cells to in vitro studies and its tendency to spontaneous activation, which can lead to artefactual results [50].

In our specific experimental conditions, we have found that caprine neutrophils are responsive against both inactivated and live *Map*, using its entire range of functions in a MOI-dependent manner. This response was stronger against live bacteria in the functions studied, being the 1:10 MOI particularly effective in eliciting this in vitro response. Within the wide variety of positive controls used in different assays (PMA, Zymosan A, LPS, saponin, etc.), two of them were selected for this study. PMA is one of the most extensively used neutrophil activator [35, 51], with a particular importance in NETosis studies [3, 50]. On the other hand, Zymosan A is an ideal subject for in vitro phagocytosis and chemotaxis tests [52], and has also been used in experiments on degranulation, superoxide production [53] and NETosis [13, 16, 52]. In this study, PMA proved to be a better positive control than Zymosan A for degranulation studies since fewer extracellular MPO + granules were observed with the former than with PMA. However, a previous study [53], detected similar levels of MPO release for both stimuli using opsonized Zymosan A at a higher concentration (3.33 mg/mL). The morphologic changes observed in PMA stimulated neutrophils are in line with those previously described, as PMA-induced neutrophil activation greatly increases cell adhesion, causing the rounding up of the cell outline and a larger appearance on microscope observation, and the exocytosis of the granules and the loose distribution of MPO granules [53]. Both PMA and Zymosan A induced the formation of NETs similar to those described previously [3, 13, 16]. However, the DNA release levels quantified through the Picogreen method using Zymosan A, do not match the NETosis observed by immunofluorescence as previously reported [16]. This could suggest that Zymosan A may contain considerable quantities of *Saccharomyces cerevisae* DNA, making it unsuitable for DNA-based NETosis quantification methods.

Regarding immunofluorescence assays, our protocol is partially based on previous works based on human [8, 35], rabbit [21] and bovine [16] neutrophils, but variations in the reagents and techniques used for fixation, washes, permeabilization, blocking and labelling, as well as its concentrations and timing have been incorporated, which leads to results not being comparable. Here in, the small NETs observed in negative controls could be attributed to spontaneous NETosis, associated with neutrophil aging-related autophagy already reported [54], and the histone-labelled nuclei most probably would be those of neutrophils in the first stages of NETosis, as chromatin decondensation would facilitate the anti-histone antibody binding. For NET quantification, although a variety of image-analysis techniques have been employed [9], they are highly subjective, particularly when using certain stimuli like mycobacteria that lead to neutrophil aggregation around clumps, making a biased counting of individual cells releasing NETs per field. In addition, although methods based on fluorescence intensity are extremely dependent on the processing, handling, and storage of both samples and reagents, with our approach based on the percentage of fields with NETs, we have tried to circumvent these drawbacks, and it has been enough to identify differences between treatments. In addition, ancillary techniques that measure the extracellular DNA have been developed to solve this problem and its simultaneous use with image-based techniques is a need. However, the methods based on DNA quantification through fluorometric methods, also present drawbacks due to the fact that some studies do not apply any mean of intra-individual normalization [55] and the cellular death, with the subsequent release of DNA, could be confused with NETosis and bias the results [56]. NETosis has been previously studied through SEM in cattle, against protozoan species [12, 13], and also in human neutrophils against *M. tuberculosis* [19], however, to our knowledge, this is the first time that this mechanism has been observed against mycobacteria in a ruminant species using this technique, and further research with other *Mycobacterium* species and study conditions could offer useful information.

In relation to the phagocytosis assay, MPO was chosen as a versatile neutrophil marker [37] for flow cytometry, as it is primarily expressed in these cells. It also serves, to some extent, as a degranulation marker, given that primary or azurophilic granules are characterized by the presence of this enzyme [57]. However, this is the first time that neutrophil MPO, and *Map*-phagocytosis levels have been measured simultaneously through flow cytometry and further studies might be needed to clarify and compare these results. In addition, the alteration of *Map* scatter parameters caused by ethanol permeabilization has proven to be a useful way to discriminate mycobacterial clumps from neutrophils by flow cytometry. This clumping problem had been previously reported [58], when working with *M. bovis* BCG, and was partially circumvented by using a lower MOI (1:1). The mechanism behind this change may lie in the extraction or alteration of mycobacterial cell wall compounds, responsible of cell aggregation [59] during the permeabilization step, in a way that changed the scatter parameters of the mycobacteria, causing the disaggregation of the bacterial clumps, as in a previous study, 80% ethanol was used to effectively extract most cell wall components from *Map* and other mycobacteria [60]. Regarding the lower GFP intensity observed in inactivated *Map*, we ascribe it to the heat-inactivation process, as GFP fluorescence intensity decreases when exposed to temperatures above 70 °C [61].

The present study has shown the strong innate response that caprine neutrophils develop against both inactivated and live *Map*, as these cells showed a tendency to form aggregates, correlated with bacterial clumps. Also, a small percentage of neutrophils phagocytose *Map*. These results are in line with those described in a similar in vitro experiment in rabbits [21], and suggest that unprimed neutrophils or at least a neutrophil subpopulation [4] have the innate ability of phagocyting this bacterium, in vitro, in a short time frame. The lower mean levels of MPO detected in neutrophils with phagocyted *Map*, could be attributed to the granule-phagosome fusion leading to MPO degradation [3], to mycobacteria inducing granule exocytosis [17] or even to these cells constituting a different neutrophil subpopulation [4]. The existence of distinct neutrophil subsets or temporary functional states has been demonstrated in several species [62], including naïve cattle [63]. Some subsets have demonstrated an increased phagocytic activity [64] and, in active tuberculosis, low density neutrophils are present in high numbers, but its role is still unknown [65], these recent findings show that the available information about neutrophils and their role in mycobacterial diseases is scarce. Regarding neutrophil degranulation in response to *Map*, it was clearly directional, with MPO + granules observed mostly over bacterial clumps. Degranulation in response to *M. leprae* has been recently demonstrated in vitro using other techniques, as well as evidence of this process occurring in vivo, in infected humans [18]. However, this function is poorly studied in mycobacterial infections, and it could be of great importance as MPO exerts an antimicrobial activity against *Mycobacterium* [66], and it is worth mentioning that MPO+ (azurophilic) granules are released last, and therefore, secretory vesicles, tertiary and secondary granules could be acting on bacteria much earlier.

Concerning NETosis, we found that live *Map*, and a higher MOI, increase the response, however, differences were of low significance, and an increase in the number of animals should reinforce any findings in future experiments. The lower response against k*Map* could be caused by the lack of metabolite secretion. Furthermore, it has been proposed that neutrophils tend to phagocyte individual mycobacteria, but release NETs against large clumps of bacteria which they are not able to phagocyte [67]. In this sense, in our experience, though small clumps were still present after inactivation, this bacterial clumping is mainly seen when using live bacteria, particularly at the 1:10 MOI, and it was in the vicinity of these bigger clumps where larger numbers of neutrophils were undergoing NETosis. So it makes sense that neutrophils employ this mechanism particularly against pathogens which, due to their size, are difficult to phagocytose, and it would explain why NETosis has been widely demonstrated in ruminants against a variety of protozoan pathogens [12, 13]. Regarding the DNA release quantification, recent research has found similar NETosis levels in bovine neutrophils using a 1:1 MOI of both live, and heat-inactivated *Map* K10 [16], however, no measurable DNA release was found in similar experiments, using a 1:10 MOI of live bacteria, in the ovine species, and only a few NETs were observed through immunofluorescence [22], this could be caused by differences in host response against *Map* between species [68].

Finally, regarding transcript expression of cytokines, a significant increase in the expression of the proinflammatory cytokines IL-1β and TNF in neutrophils exposed to *Map* at high MOIs was observed. Specifically, IL-1β is critical for neutrophil migration [69], necessary for the production of NETs [70]. Additionally, it has an important role in the orchestration of the adaptative immune response, regulating the expansion and effector function of T lymphocytes [10]. On the other hand, TNF enhances phagocytic properties of neutrophils [71], induces NETosis [72] and primes neutrophil granule exocytosis [73]. TNF expression and production by neutrophils is increased in response to *M. tuberculosis* in vitro [74] and, an increase in IL-1β, attributed to neutrophil production, has been described in neutrophilic granulomas induced by *M. marinum* [75], findings that highlight the importance of these cytokines in mycobacterial infections. The role of these cytokines in *Mycobacterium* infections has not only been confirmed in the simplistic microenvironment of in vitro settings, but also in in vivo studies, as both TNF and IL-1β KO mice are extremely sensitive to *M. tuberculosis* infection [76], and an increase in the expression of both cytokines has been described in intestinal tissues of paratuberculosis infected sheep [77]. The production and secretion of mature IL-1β requires the prior activation and assembly of the inflammasome complex, and the initiation of a proinflammatory response, and TNF may mediate in the activation of this inflammasome [78], whereas IL-1β augments TNF expression, at least in lung epithelium [79]. On the other hand, TGF-β is a traditionally anti-inflammatory cytokine but, in the presence of IL-6 is also involved in the Th17 immune response [80]. The absence of significant changes in TGF-β expression in neutrophils exposed to *Map*, is in agreement with what has been observed in vivo, as no change in TGF-β expression was detected in paratuberculosis infected intestinal tissues [77]. It has to be taken into account that studies on neutrophil cytokine expression against mycobacterial infections in ruminants are scarce, particularly against *Map*, but the data here in obtained were enough to show a proinflammatory pattern in cytokine expression, that could modulate the local immune response in the early phases (2–12 h) of the disease, as abundant neutrophils have been identified in the first hours of *Map* infection in the bovine ileum, as well as an increase in both TNF and IL-1β [28].

Overall, if adequately timed, the rapid response of neutrophils could contribute to the control of *Map* infection, in its initial stages, directly, through its effector mechanisms (NETosis, degranulation, phagocytosis), as proposed for *M. tuberculosis* [25] and indirectly, as efferocytosis of the antimicrobial compounds involved in the initial neutrophil response could reduce the risk of macrophage colonization [16]. Additionally, the proinflammatory profile of cytokine production could shape a more effective adaptive response and further contribute to the control of *Map* immune evasion and proliferation.

To conclude, these results confirm that, the study of different neutrophil effector mechanisms, is essential to reach a better understanding of host-pathogen interactions. A comprehensive approach, with a combination of, ideally, standardized, and complementary techniques, should be employed when studying neutrophil functions. Despite the intrinsic difficulties of working with neutrophils, the applied set of techniques, has allowed us to study neutrophils’ most important functions and to demonstrate that they are highly responsive against *Map*. However, additional studies for quantifying the bacterial killing capacity of caprine neutrophils, measure the generation of ROS, central mechanism in the neutrophil response, directly implicated in all the studied functions, as well as assays on the enzymatic activity or cell signaling, should be also used in further studies. Nevertheless, these results could set the basis for future work focused on the early neutrophil response against *Map* and the effect that vaccination could have on it. However, it should be noted that these preliminary results came from a small number of similarly aged animals from only one goat breed and from the same flock, and therefore they should be taken with caution, considering the genetic variations in the overall population and other confounding factors. Also, they should be studied in vivo, on intestinal and lymphoid tissues, a much more complex environment where this response would be modulated by other immune cells, cytokines and other cell signaling molecules that cannot be mimicked in in vitro conditions.

## Supplementary Information

**Additional file 1. Sequences of primers used for qRT-PCR and standard curve data.**

**Additional file 2. Scanning electron microscopy micrographs of non-stimulated (control) neutrophils. A**, **B** Neutrophils with uniform shape and sizes can be seen distributed throughout the sample and do not show signs of activation or cell death.

## Data Availability

The data supporting the conclusions of this article will be made available by the authors, under reasonable request.
