# Supplementary material for: Evaluation of the innate immune response of caprine neutrophils against Mycobacterium avium subspecies paratuberculosis in vitro
Source: Vet Res. 2023 Jul 18;54:61. doi: 10.1186/s13567-023-01193-7 (PMC10355032; doi:10.1186/s13567-023-01193-7)
Supplement: Supplementary file 1 — Additional file 1. Sequences of primers used for qRT-PCR and standard curve data. [file 13567_2023_1193_MOESM1_ESM.docx]

Additional file 1

| **Target^a^** | **Primer** | **Primer sequences (5’-3’)** | **Product size (bp)** | ***R*^2 b^** | **Slope^c^** | **Efficiency (%)** | **Reference** | |
| --- | --- | --- | --- | --- | --- | --- | --- | --- |
| TNF (NM_001024860.1)^d^ | QTNFα-Fw | CCAGAGGGAAGAGCAGTCC | 126 | -3.77 | 0.992 | 84.2 | [38] | |
|  | QTNFα-Rv | GGAGCGCTGATGTTGGCTAC |  |  |  |  |  | |
| IL-1β (NM_001009465.2) | QIL1β-Fw | ACCCCAAAGTCTACCCCAAG | 99 | -3.58 | 0.977 | 90.2 | [38] | |
|  | QIL1β-Rv | TGAGTCTGTCCTGTACCCTA |  |  |  |  |  | |
| TGF-β (NM_001009400.1) | QTGFb-Fw | GGTGGAATACGGCAACAAAA | 117 | -3.615 | 0.951 | 89 | [39] | |
|  | QTGFb-Rv | CGAGAGAGCAACACAGGTTC |  |  |  |  |  | |
| β-actin (NM_001009784.1) | QBACTIN-Fw | ACACCGCAACCAGTTCGCCAT | 216 | -3.741 | 0.953 | 85.1 | [38] | |
|  | QBACTIN-Rv | GTCAGGATGCCTCTCTTGCT |  |  |  |  |  | |
| SDHA  (NM_174178) | QSDHA-Fw | ACCTGATGCTTTGTGCTCTGC | 126 | -3.559 | 0.998 | 90.97 | [40] | |
|  | QSDHA-Rv | CCTGGATGGGCTTGGAGTAA |  |  |  |  |  | |
| GADPH  (JN811680.1) | QGADPH-Fw | CTGGCCAAGGTCATCCAT | 86 | -3.325 | 0.994 | 99.84 | [41] | |
|  | QGADPH-Rv | ACAGTCTTCTGGGTGGCAGT |  |  |  |  |  | |
| ^a^ NCBI accession numbers are for *Ovis aries* or *Capra hircus* cDNA sequences used in primer design. When using ovine sequences, they were checked for similarities with caprine sequences using basic local alignment search tool BLAST  ^b^ Mean minimum coefficient of regression (R2) of standard curves  ^c^ Mean of standard curve slopes.  ^d^ GenBank accession number. | | | | | | | |  |
