# Supplementary material for: Evaluation of the innate immune response of caprine neutrophils against Mycobacterium avium subspecies paratuberculosis in vitro
Source: Vet Res. 2023 Jul 18;54:61. doi: 10.1186/s13567-023-01193-7 (PMC10355032; doi:10.1186/s13567-023-01193-7)
Supplement: Supplementary file 2 — Additional file 2. Scanning electron microscopy micrographs of non-stimulated (control) neutrophils. A, B Neutrophils with uniform shape and sizes can be seen distributed throughout the sample and do not show signs of activation or cell death. [file 13567_2023_1193_MOESM2_ESM.docx]

**Additional file 2**

**
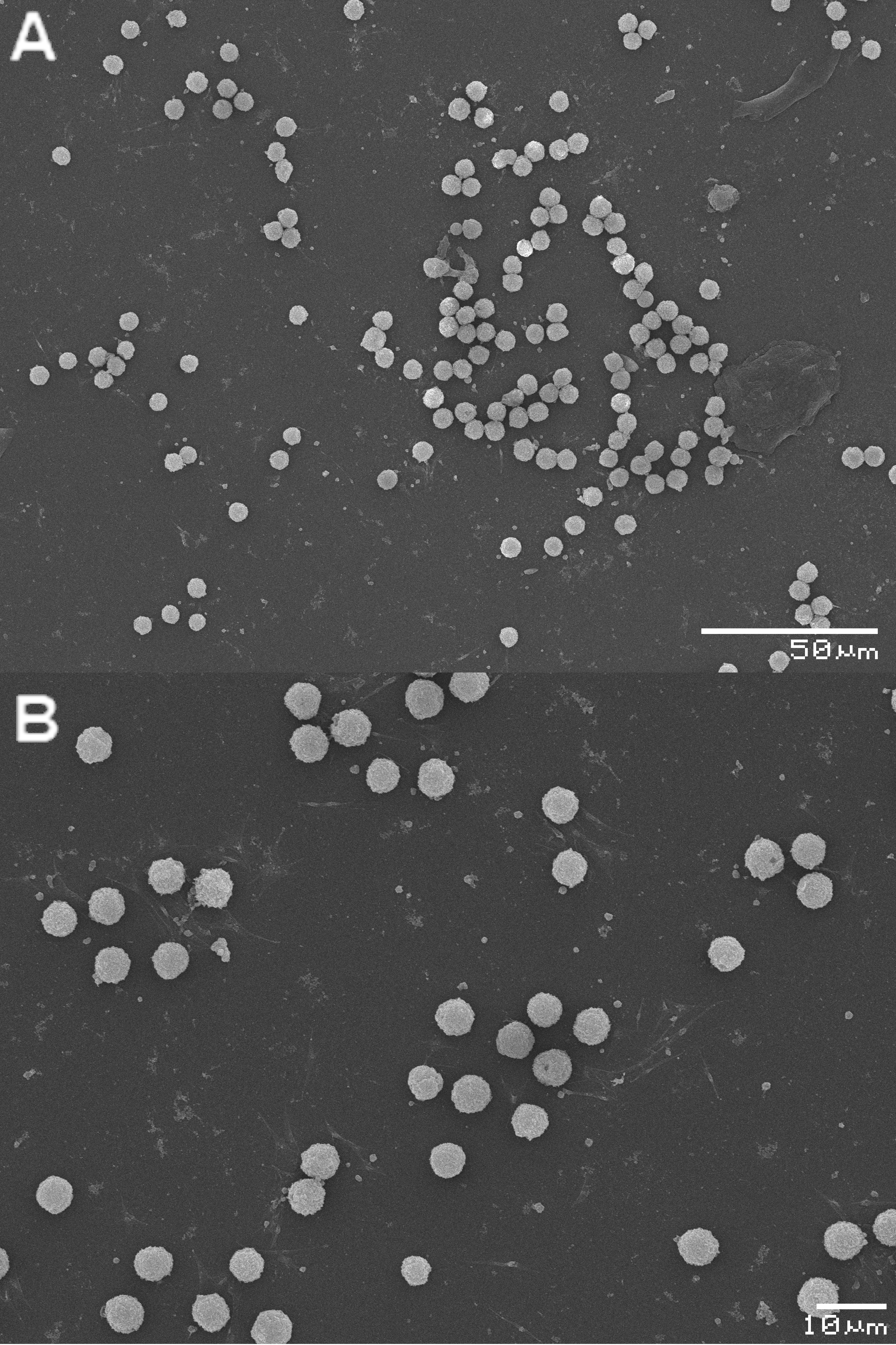
**

**Scanning electron microscopy micrographs of non-stimulated (control) neutrophils. (**A, B) Neutrophils with uniform shape and sizes can be seen distributed throughout the sample and do not show signs of activation or cell death.
